# Visual Exploration of Dynamic or Static Joint Attention Bids in Children With Autism Syndrome Disorder

**DOI:** 10.3389/fpsyg.2019.02187

**Published:** 2019-10-09

**Authors:** Federica Cilia, Alexandre Aubry, Barbara Le Driant, Beatrice Bourdin, Luc Vandromme

**Affiliations:** ^1^EA7273 Centre de Recherche en Psychologie Cognition, Psychisme et Organisations, Université de Picardie Jules Verne, Amiens, France; ^2^EA7489, Laboratoire Lorrain de Psychologie et de Neurosciences de la Dynamique des Comportements (2LPN), Institut National Supérieur du Professorat et de l'Éducation, Université de Lorraine, Nancy, France; ^3^EA 7516, Chirurgie et Extrémité Céphalique, Caractérisation Morphologique et Fonctionnelle, Université de Picardie Jules Verne, Amiens, France

**Keywords:** stimuli, joint attention, autism spectrum disorder, eye-tracking, areas of interest (AOI)

## Abstract

Eye-tracking studies have revealed a specific visual exploration style characterizing individuals with autism spectrum disorder (ASD). The aim of this study is to investigate the impact of stimulus type (static vs. dynamic) on visual exploration in children with ASD. Twenty-eight children with ASD, 28 children matched for developmental communication age, and 28 children matched for chronological age watched a video and a series of photos involving the same joint attention scene. For each stimulus, areas of interest (AOI) were determined based on Voronoi diagrams, which were defined around participants' fixation densities, defined by the mean shift algorithm. To analyze the eye-tracking data on visual exploration, we used a method for creating AOI *a posteriori*, based on participants' actual fixations. The results showed the value of both kinds of stimuli. The photos allowed for the identification of more precise AOI and showed similarities in exploration between ASD and typical children. On the other hand, video revealed that, among ASD children only, there are few differences in the way they look at the target depending on the deictic cue used. This raises questions regarding their understanding of a joint attention bid recorded on a video. Finally, whatever the stimulus, pointing seems to be the most important element for children looking at the target.

## Introduction

Children with autism spectrum disorder (ASD) show atypical visual behavior (Senju et al., 2008; Jones and Klin, 2013). Eye-tracking devices make it possible to investigate these children's social skills without any need for verbal instructions. Eye-tracking has provided results that show how children with ASD visually explore images (e.g., Riby and Hancock, 2008; Freeth et al., 2010) and videos (e.g., Klin et al., 2002; Nakano et al., 2010; Jones and Klin, 2013), as well as their visual exploration during direct interactions (e.g., Navab et al., 2012; Noris et al., 2012). Most of the time, the data used comes from areas of interest (AOI) drawn up *a priori* by the researchers.

Several studies question visual exploration by using different types of gaze following stimuli or joint attention stimuli. Visual attention is an essential element of joint attention, which corresponds to the subject's ability to coordinate their attention with that of the partner in order to share a common experience related to an object or an event. Joint attention involves referential gazing, pointing, and verbalization (Mundy et al., 2009). Like the gaze monitoring task developed by Scaife and Bruner in 1975, most of the studies that have questioned joint attention over the past 40 years use attention cueing paradigms in order to operationalize joint attention (Aubineau et al., 2015, for review). Attention cueing paradigms were defined as response to joint attention (Mundy et al., 2009). Jording et al. (2018) introduced the “Social Gaze Space,” which represents all the internal states observed in gaze-based triadic interactions. There are different categorical states: “partner-oriented,” “object-oriented,” “introspective,” “initiating joint attention,” and “responding joint attention.” Regarding the joint attention response, Jording et al. (2018) explain that, in literature, “the invitation of another person thereby establishing a rudimentary form of joint attention appears to be deeply rooted in human behavior.” This can be observed in a naturalistic interaction implying that “B chooses an object and A follows B's gaze toward the object,” which leads us to the conclusion that the same process is operationalized in joint attention tasks. Therefore, most eye-tracking studies of joint attention in literature have been limited to an analysis based on tracking the gaze line, with or without head orientation (Riby and Doherty, 2009; Bedford et al., 2012; Falck-Ytter et al., 2012, 2015; Billeci et al., 2016), or on visual tracking of pointing (Falck-Ytter et al., 2012; Benjamin et al., 2014; Franchini et al., 2017). In addition, the AOI used are frequently the face and/or the referent, also called the “target” (Swanson et al., 2013; Franchini et al., 2017), as well as the hand that is pointing at the target (Benjamin et al., 2014).

During eye-tracking tasks, children with ASD were less likely than typically developing children, to look at faces. This is perceptible when confronted with a static (Riby and Doherty, 2009) or a dynamic stimulus (Franchini et al., 2017). In fact, they tend to prefer looking at the background of the stimuli, that is to say, elements outside AOI, also called “white space.” Such attention to white spaces is also observed on a static (Riby and Hancock, 2008; Chawarska and Shic, 2009) or dynamic stimulus (Nakano et al., 2010). When dynamic stimuli such as videos are used, toddlers with ASD are able to follow the direction of a gaze toward an attentional target (Bedford et al., 2012; Billeci et al., 2016). Conversely, children with ASD aged about 11 years old do not always follow an actor's gaze to the correct target on static stimuli such as photographs (Riby et al., 2013; Billeci et al., 2016). In addition, children with ASD aged about seven take more time than typical developing children to follow an actor cue on static stimuli (Riby and Doherty, 2009).

Despite the differences from one study to another, few eye-tracking studies have compared fixations data in static and dynamic stimuli. Hanley et al. (2013), using only photographs, show differences between static faces and dynamic scenes involving social interactions. The results show that when children with ASD look at a social scene, they have difficulty looking at actors' eyes. Nevertheless, few studies have compared eye-tracking data gathered from children with ASD who have been engaged with both static (such as photos) and dynamic stimuli (such as videos). To the best of our knowledge, only three studies using modern eye-tracking equipment have made a comparison between static photographs and dynamic videos (Saitovitch et al., 2013; Shic et al., 2014; Chevallier et al., 2015). All three studies showed an effect of stimulus type (dynamic vs. static) on the visual behavior of children with ASD. Shic et al. (2014) presented stimuli to 6-month-old babies at risk of developing ASD. They presented three types of percepts (images, a video of a female actress smiling, and a video of a female actress smiling and speaking in motherese). In this study, the authors found that children at risk of developing ASD paid remarkably less visual attention to the faces. When they looked at the stimuli, they were less likely to look at items providing social information, such as the face of the actor speaking. Finally, the authors observed an effect of stimulus condition regarding fixations on the eye region, which was less distinct in the case of the dynamic stimulus where the actress was speaking compared to the other stimuli. Saitovitch et al. (2013) investigated the effect of stimuli used in eye-tracking studies on children with ASD. They used four kinds of stimuli to this end: a video with human actors, a cartoon video, photos of human actors, and photos of cartoon characters. They compared the number of fixations on different AOI defined *a priori*. The obtained results showed that children with ASD were more focused on the background when looking at videos than when looking at photographs. However, they did not find any significant differences among the different kinds of stimuli in social AOI such as faces, particularly within the eye and mouth regions. Finally, Chevallier et al. (2015) presented static and dynamic visual exploration tasks to subjects with ASD aged 6 to 17. The static task comprised about 10 items including objects and faces. In the dynamic exploration task, the children saw, in a single stimulus, two videos showing faces expressing emotions, and two videos showing objects. Finally, the authors administered a dynamic exploration task of a more natural scene showing siblings playing together. Only this last task enabled the differentiation of children with ASD from the control group. In fact, ASD children spent less time looking at the social stimuli and more time looking at the non-social aspects of the scene than the control group children. Thus, these studies showed that, regardless of the stimulus type, visual exploration by children with ASD of AOI, such as the eyes or mouth, remained unchanged.

Although the three studies described above focused on social situations, none of them examined visual behavior in joint attention bids. Moreover, all three studies were based on an analysis of AOI defined *a priori*. In most studies of autism that used eye-tracking, visual behavior is analyzed on the basis of AOI predefined by experimenters in a top-down approach (e.g., Jones et al., 2008; Chawarska and Shic, 2009; Shic et al., 2014; Franchini et al., 2017). Furthermore, how these *a priori* AOI are constructed is more often than not indicated in studies (Hessels et al., 2016). Thus, the data resulting from *a priori* AOI are limited to fixations and saccades observed in these areas, as a function of their size and arrangement, as predefined by experimenters (Hessels et al., 2016). This subjective method of defining AOI has consequences for the comparison of different eye-tracking studies. In addition, young children's fixations can be very dispersed, particularly if they have certain developmental disorders such as ASD.

To the best of our knowledge, few psychology studies have integrated a method for identifying AOI based on participants' visual activity, in other words, a bottom-up approach (Liberati et al., 2016). One of the objectives of this article is to introduce the Voronoi method based on Voronoi diagrams (see Over et al., 2006, for details). This method makes it possible to create AOI corresponding to cells defined around participants' different fixation densities identified by mean shift clustering (e.g., Privitera and Stark, 2000; Santella and DeCarlo, 2004; Duchowski et al., 2010; Einbeck, 2011; Drusch and Bastien, 2012; Krejtz et al., 2013). This bottom-up approach has the advantage of using the entire distribution of participants' visual fixations and defining the coordinates of the different fixation density centers. Thus, the experimenter does not choose the size and location of AOI. Cilia et al. (2019) compared *a priori* and *a posteriori* AOI data. They observed that children look less at the joint attention target with *a priori* analysis of the data, compared to the Voronoi diagram method. Moreover, with *a priori* AOI, children with typical development focus more on faces than ASD children, while with the *a posteriori* method, there is no difference between the two groups.

### The Current Study

The main objective of this study is to compare the visual behavior of children with ASD, depending on the nature of the stimulus (dynamic vs. static) in situations involving joint attention. Based on literature, we hypothesized that children with ASD would focus more on backgrounds rather than on faces, compared to typical children when confronted with a static stimulus. On the other hand, when confronted with a dynamic stimulus, we expected both groups to concentrate more on faces rather than on backgrounds. Moreover, we expected ASD children to focus more on the dynamic stimulus rather than on the static stimulus. Finally, we hypothesized that the number of deictic cues (looks, pointing, verbalizations) used to show the target would have an impact on the duration of visual fixation of the target.

## Materials and Methods

### Participants

Thirty children with ASD took part in the study. Two ASD participants were excluded because they had not done the socio-communicative evaluation. The final sample was composed of 28 children with ASD, aged from 31 to 147 months (M = 90.43 months, SD = 31.88 months). The socio-communicative development of the children was evaluated with the French version of the Early Social Communication Scale (ECSP, Guidetti and Tourrette, 2009). The children with ASD had a communicative developmental age from 8.0 to 31.0 months (M = 24.07 months, SD = 7.15 months). The diagnosis of ASD was made by a psychiatrist in the Hauts-de-France region on the basis of various French version of standardized tools (Autism Diagnostic Interview–Revised, ADI-R, Lord et al., 1994; and/or Autism Diagnostic Observation Schedule–Generic, ADOS-G, Lord et al., 2000). However, we did not get permission to read the children's medical files. ADI-R and ADOS-G scores were not analyzed in this study. They were treated in medical and/or social institutions in the Hauts-de-France region (e.g., day hospital, clinic for children with developmental disabilities), but these children did not participate in any specific training program for joint attention.

Fifty children were also included in the control group. Twenty-eight children aged from 43 to 154 months (TC: M = 95.32 months, SD = 35.92 months) were matched to the ASD children in terms of chronological age. Twenty-eight children aged from 10.00 to 33.00 months matched the ASD children in terms of socio-communicative developmental age only. According to their results at the ECSP, there was no gap between the actual age of TD children and their developmental level of socio-communicative abilities. The control group children had no siblings with ASD, and no proven developmental disorders. None of the children showed hearing or visual impairment or had a known genetic syndrome. Table 1 presents the characteristics of the sample.

**Table 1:** Participants' characteristics.

	**ASD** ***n*** **=** **28**		**TD** ***n*** **=** **25**		**TC** ***n*** **=** **25**	
	**M**	**SD**	**M**	**SD**	**M**	**SD**
Chronological age (years, months)	7, 7	2, 7	2, 2	0, 7	7, 8	2,11
Developmental age on the ECSP (months, days)	24, 10	7, 8	23, 15	6, 7	–	–
Score total ECSP	139.95	50.8	139.18	49.5	–	–

All children's parents and/or legal guardians were informed of the objectives of the study, the nature of the tasks that would be administered, and the fact that they could withdraw their agreement at any time. Their informed consent was received in writing in accordance with the Declaration of Helsinki of June 1964 (amended at the 64th General Assembly of the World Health Organization in October 2013). Moreover, all children gave their agreement to participate, and if they wished, parents could be present near their children in the experimental room. This study did not require authorization from the ethics committee, based on the recommendations for psychological research in France and in agreement with the national and institutional guidelines (https://www.legifrance.gouv.fr/eli/decret/2017/5/9/AFSP1706303D/jo/~texte).

### Apparatus and Stimuli

The study was carried out with an SMI-RED 250 Hz eye-tracker (SensoMotoric Instruments, SMI) calibrated at 60 Hz. Data and stimuli were recorded in SMI's *I View X* and *Experiment Center* software, respectively. They were presented on a 17” screen in 4:3 format, the dimensions of which were 34.7 × 25.9 cm. Eye movements were recorded binocularly. The raw data were extracted with *Begaze* software and fixations were classified using the *Gazepath* package (van Renswoude et al., 2018) on R. The *Gazepath* package takes into account data from both eyes. It averages the *x* and *y* coordinates and interpolates the missing data based on the data of the other eye. The velocity threshold is estimated using the same method as Mould et al. (2012). It is estimated by the distance between the preceding and succeeding points, divided by the time elapsed between them on a trial-by-test basis for each individual. This allowed for the calculation of the speed of the eye that corresponds to the Euclidean distance between two points divided by the time line. If the data exceed the usual speed of the eye, it is classified as a saccade; otherwise, it is a fixation. According to the literature, saccades are worth about 200 ms (Nyström and Holmqvist, 2010). When there are successive fixations that follow each other, the package made a correction. In this case, the fixations are merged into one. Finally, the duration velocity thresholds calculated participant by participant that were greater than the optimum duration threshold (that represents the noise) were classified as fixations. After classifying the data as fixations, *a posteriori* AOI were created with the *ks* package (Duong, 2007) for the mean shift algorithm, and the *deldir* (Turner, 2018) and *sp* (Bivand et al., 2013) packages for the Voronoi Tessellation method. All statistical pre-analyses and analyses were done using *R* software (R Core Team, 2016). Our stimuli and the R script with all the functions allowing the analysis of raw eye-tracker data are available on the Open Science Framework (OSF) platform (https://osf.io/8ewsk/) and are explained in detail in a French article (Cilia et al., 2019).

The material consisted of a video presented to a raster width of 1,280 pixels and a raster height of 1,024 pixels. A 2 cm-thick black band outlined the stimulus. The audio transmission was at 160 kbit/s. The video image rate was 25 frames per second. The data that were used came from the first 20.3 s of a 58 s video from which we extracted five video sequences with a duration of 1.21 to 2.9 s. In addition, five photos taken as screenshots from the video were presented for the same duration as their corresponding videos. The scene presented in the video and corresponding photos started with an attention grabber to the child (i.e., a cartoon hand waving) to attract their attention. Then the woman in the video said “hello, how are you?” to the child and looked and/or verbalized or pointed and/or verbalized at a joint attention target. We did not analyze a large part of the video and photos. Indeed, doing so was a possible answer to another research question about a target absent from the visual field of the child (shown by the adult by the same deictic clues: looks, pointing, verbalizations). For half of the children, the target was non-contingent with the previous target; for the other half, the target was contingent with the clues. That is why, in the part of stimulus we are analyzing here, there is no competitive target.

### Procedure

During the task, depending on their age and motrice agitation, children were seated comfortably approximately 60 cm from the screen, on a chair, on a parent's lap, or in a highchair. The experimental area was as uncluttered as possible to avoid any distractions. In fact, children faced the screen and were surrounded by two white curtains. Behind each child, the parent or experimenter helped them maintain the optimal position for data recording. During the experiment, the only instruction the children were given was “Look at the screen,” which may have been repeated several times. Babies in the control group and children with autism were not verbal or did not exceed the level of children aged 30 months. Therefore, we did not give them any instructions. Children were invited to sit down and watch the computer screen. For matched children in chronological age, we explained the purpose of the study and told them that they may find the videos and images childish. We specified that they did not need to remember the images; they only needed to watch the screen.

After a five-point calibration phase, a video and photos were presented to the children in a counterbalanced way in the course of a wider-ranging research protocol. The order of presentation of the stimuli was counterbalanced with other stimuli (i.e., gaze following videos, photos of faces or objects, inscribed in a larger research protocol). The experiment in eye-tracking lasted 7 min in total. Regarding this part, children saw two photographs and two videos where the joint attention target was located to the left or right of the actor. The script involved the following situations: the actress had her mouth open and one hand moving in a greeting gesture; in the video, the actress said “*coucou, coucou, tu me vois”* (“hey, hey, you see me”), gesturing with her hand to attract attention (we offer no analysis of this situation). (1) The first sequence we analyzed shows the actress facing and looking at the camera. (2) In the second, the actress turned her eyes to look at the target. (3) Then, the actress turned her head to look at the target (we see her in profile). (4) In the fourth sequence, she is still in profile but, additionally, points at the target. (5) Finally, in addition to the precedent sequence, she speaks about the target, saying “*Oh regarde!”* (“Oh, look!”).

We took five screenshots of this video and showed them to the children. In correspondence with the rest of the protocol, we chose not to make children listen to music while they were looking at the photos. Moreover, to prevent children from thinking the photos are “weird,” while they were looking at them, we chose not to add sound to these photos. This way, when the children were not attentive, the experimenter would say “look at the screen.”

### Coding and Analysis

This article analyzes visual exploration on the basis of AOI created *a posteriori* using the Voronoi Tessellation method (Over et al., 2006) based on fixation densities estimated with mean shift clustering (Santella and DeCarlo, 2004). First, raw data were analyzed with the *Gazepath* package (van Renswoude et al., 2018). Mould et al. (2012) algorithm was used to classify fixations and saccades. The advantage of this algorithm is that it sets different detection thresholds based on the quality of the raw data. The lower the quality of the data, the more conservative the thresholds used (see van Renswoude et al., 2018, for details).

Next, the mean shift algorithm enabled us to identify different fixation densities for all children for each photo and corresponding video sequence. Thus, we obtained coordinates for each participant's visual fixation densities on a Euclidean plane. Generally speaking, mean shift clustering consists in finding the centroid of the data density by creating a vector for each point that will contribute to the local mean. Each centroid makes it possible to identify the distribution density kernel or core. Finally, the algorithm is able to identify clusters, based on the convergence degree of the coordinates in the *x* and *y* positions of each fixation point in relation to the density kernel.

Finally, we used Voronoi's method (Over et al., 2006) to create AOI that were large enough to be less sensitive to typical eye-tracker errors (Hessels et al., 2016). This method is used to divide areas centered around a number of points. Each cell represents the area that is closest to one of the points, and lines indicate equidistant locations between points or cell centers. Shape and size of *a posteriori* AOI are more objective than *a priori* AOI thanks to a machine that takes into account all the parts of the stimulus (Hessels et al., 2016). Then, AOI were named by the experimenters depending on the location of the different cells on the image (i.e., video and photo). These *a posteriori* AOI corresponded to different parts of the stimulus that had attracted the participant's visual attention, such as the face or the joint attention target. This method can be used with all kinds of stimuli, since it is based on recorded data.

Our analyses tested the effects of group, AOI, stimulus condition and stimulus type, and interaction effects. We chose to investigate a dependent variable found in the literature (Guillon et al., 2014; Cilia et al., 2018): relative fixation duration (RFD), which considers the duration of fixations relative to each child's total fixation duration on the screen. Thus, RFD shows the amount of time spent fixating one area compared to the rest of the screen.

### Statistical Analyses

All data analyses and processing were performed using R 3.6.0 (R Core Team, 2016). All data and R scripts are available on the Open Science Framework (OSF) platform (https://osf.io/8ewsk/). Statistical analyses were performed based on the general linear model, using mixed-design ANOVAs (with Greenhouse-Geisser correction when appropriate). Subsequent comparisons were conducted using Tukey's HSD with Holm's correction for each condition and group when appropriate. Effects sizes were computed using partial eta squared. The normality and homoscedasticity assumptions were checked graphically. The influence of potential outliers was estimated using Cook's distance. According to the recommendations of Aguinis et al. (2013), we have determined Cook's distance cutoff using the *F* distribution with df1 = *k* − 1, df2 = *k* − *n* − 1, and α = .50, where *k* is the number of predictor and *n* is the size of sample (Cohen et al., 2003).

## Results

### Qualitative Results

**Figure 1:**
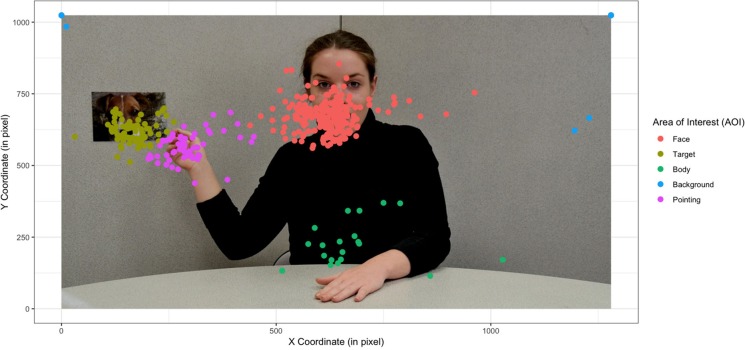
Representation of fixation centroids independently of group and stimulus type (static vs. dynamic) during a joint attention bid involving a pointing action. The actress was informed and gave her written consent for scientific publication of her image.

In a developmental psychopathology–based approach, it is essential to start from the specific features of the visual exploration of an atypically developing child. In this study, it is therefore legitimate to wonder which areas of the screen the children looked at preferentially. One might wonder whether there is really an AOI on the pointing hand, the eyes, and the mouth, depending on the stimulus type (dynamic vs. static). The qualitative results show an impact of the stimulus. Given the size of the image and the precision of the eye-tracker, it was impossible to separate the data on the eyes and the mouth. A more general face AOI was created for all groups and for both stimulus types (i.e., video and photo). On the other hand, the mean shift algorithm identified a fixation density more easily on the hand of the actress when she was pointing at the target in static than in case of dynamic stimuli (see Figure 1).

**Figure 2:**
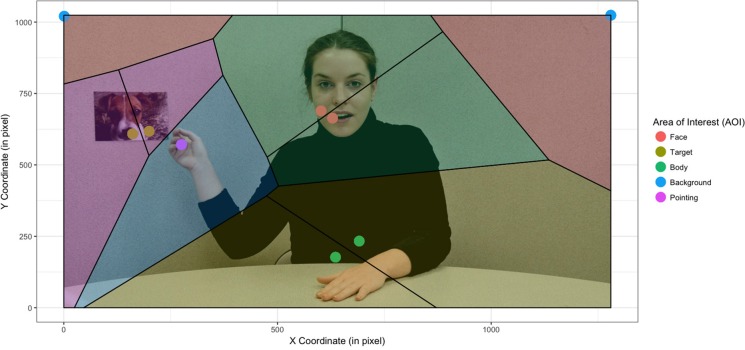
Creation of cells by the Voronoi method based on the fixation centroids independently of group and stimulus condition (static vs. dynamic). The actress was informed and gave her written consent for scientific publication of her image.

An initial conclusion on the effect of stimulus was therefore that stimulus does have an impact on clustering and thus an impact on the AOI from which we would extract our data. From a quantitative perspective, we opted to use not only mean shift clustering but also Voronoi's method, which allowed us to divide the screen into AOI that were sufficiently large for all the clusters of the different groups of children to belong to the same AOI (see Figure 2).

### Quantitative Results

#### Intergroup Analyses

A 3 (Group: ASD vs. TC vs. TD) × 2 (Condition of stimuli: static vs. dynamic) × 3 (AOI type: Background vs. Face vs. Target) mixed ANOVA was performed.

For target fixation in each joint attention stimuli, a 3 (Group: ASD vs. TC vs. TD) × 2 (Condition of stimuli: static vs. dynamic) × 5 (Stimulus) mixed ANOVA was performed on RFD.

A significant main effect of group shows that each group did not have the same rate of fixations on average, *F*_(2, 81)_ = 6.31, *p* < 0.001, η*p2* = 0.03. Independently of condition and AOI, *post-hoc* comparison using Tukey HSD indicates that the fixation duration of children with ASD (M = 0.120, SD = 0.154) is lower on average than that of children in the TC group (M = 0.154, SD = 0.202), but there is no difference between the ASD and the TD group (M = 0.143, SD = 0.17). There was a significant main effect of condition, *F*_(1, 81)_ = 30.70, *p* < 0.001, η*p2* = 0.04, and there was a significant interaction between group and condition, *F*_(2, 81)_ = 3.61, *p* < 0.001, η*p2* = 0.01. Children in the TC group and in the TD group made longer fixations when confronted with static stimuli (TC: M = 0.177, SD = 0.211, TD: M = 0.166, SD = 0.205) than when confronted with dynamic stimuli (TC: M = 0.131, SD = 0.191, TD: M = 0.121, SD = 0.146). Only children with ASD did not differ on the duration of fixations between static (M = 0.126, SD = 0.159) and dynamic (M = 0.115, SD = 0.149) stimuli.

Moreover, a significant effect of AOI was found, *F*_(2.64, 211.15)_ = 172.60, *p* < 0.001, η*p2* = 0.19, and a significant interaction between the group and AOI has shown specific gaze behaviors in children in each group, *F*_(5.28, 211.15)_ = 5.50, *p* < 0.001, η*p2* = 0.015. Through this significant interaction effect, all children had a similar pattern on gaze behaviors in function of the type of AOI. Indeed, *post-hoc* comparison using the Tukey HSD test has shown that children fixations are longer on “Face” AOI (M = 0.349, SD = 0.383) than on “Target” AOI (M = 0.205, SD = 0.314), on “Face” AOI than on “Background” AOI (M = 0.043, SD = 0.174), on “Target” AOI than on “Background” AOI.

**Figure 3:**
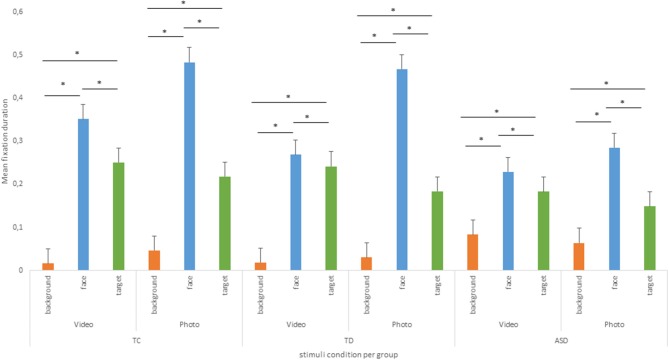
Mean of relative fixation duration by group and AOI. * *p* < 0.05.

Finally, there was a significant interaction between stimulus and AOI, *F*_(2.77, 221.80)_ = 14.15, *p* < 0.001, η*p2* = 0.014. Through this significant interaction effect, all children had a similar pattern on gaze behaviors in function of the stimulus condition. When they watched videos, they made longer fixations on “Face” AOI (M = 0.284, SD = 0.169) than on “Target” (M = 0.226, SD = 0.153) and on “Background” AOI (M = 0.039, SD = 0.095), and they made longer fixations on “Target” AOI than on “Background” AOI. In the same way as when they watched photos, they made longer fixations on “Face” AOI (M = 0.414, SD = 0.191) than on “Target” AOI (M = 0.184, SD = 0.135) and on “Background” AOI (M = 0.047, SD = 0.950), and they made longer fixations on “Target” AOI than on “Background” AOI (see Figure 3).

**Figure 4:**
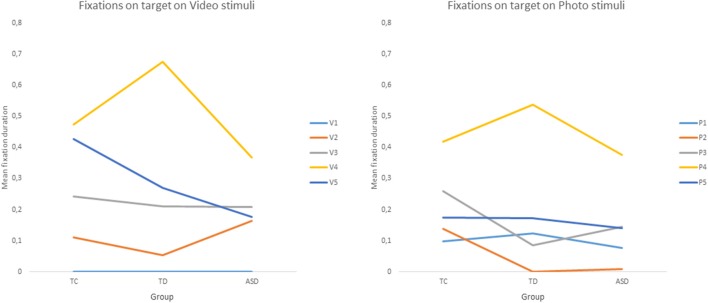
Mean of relative fixation duration on target by group and stimuli. *Legend*: V1, V2, V3, V4, V5 = Video stimuli; P1, P2, P3, P4, P5 = Photo stimuli.

Concerning the target analysis, there was a significant main effect of condition *F*_(1, 0.36)_ = 5.031, *p* < 0.005, η*p2* = 0.06. Children fixated on the target for longer in videos (M = 0.226, SD = 0.342) than in photos (M = 0.184, SD = 0.281). Moreover, there was a significant main effect of stimulus, *F*_(3.52, 281.52)_ = 67.98, *p* < 0.001, η*p2* = 0.24. There was a significant interaction between condition and stimulus, *F*_(2, 0.28)_ = 0.196, *p* < 0.001, η*p2* = 0.02. Strikingly, there was a significant interaction between group and stimulus, *F*_(7.04, 281.52)_ = 3.78, *p* < 0.001, η*p2* = 0.03. *Post-hoc* tests on video stimuli have shown common points and differences between ASD children and the control groups. No child looks at the target on the first stimulus but all children look more at the target on stimulus 4 (head orientation and pointing) than on stimulus 2 (eyes orientation only). There is no other difference between stimuli for the ASD group. Among the control group, there were other differences between stimuli. Their fixations were longer on the target AOI on stimulus 4 than on stimulus 3. For the TD group, there were longer fixations on stimulus 4 than on 5 while there was no difference for the TC group. *Post-hoc* tests on photo stimuli for the ASD and TC groups have shown longer fixation on stimulus 4 than on stimulus 1, 2, 3, and 5. For TDs, there were longer fixations on stimulus 4 than 1, 3, and 5. Finally, for TCs only, fixations were longer on stimulus 5 than on stimulus 2 and 3 (see Figure 4).

## Discussion

The objective of this study was to compare visual fixation data for children with ASD as a function of stimulus type: videos and photos. Our stimuli presented joint attention scenes indicated by gaze, pointing, and verbalization (Mundy et al., 2009). To analyze fixations, we used the mean shift clustering method (Santella and DeCarlo, 2004; Einbeck, 2011), which makes it possible to identify AOI appropriately. Thus, we took into consideration eye-tracker measurement errors and eye deviations that sometimes result in imperfect calibrations. The qualitative results justified our interest in creating AOI *a posteriori* to analyze the fixation results. In our data, the main density zones and the resulting clusters are located around the face and the joint attention target. These are essential elements in a joint attention episode, and these areas have in fact been studied a good deal in the literature (e.g., Swanson et al., 2013; Franchini et al., 2017). The qualitative results showed the value of photos, since AOI in photos are more precise than in the corresponding videos. Photos allow for the *a posteriori* creation of more precise AOI, which is essential when specific factors are being examined such as the addition of a visual cue to the joint attention scene (e.g., orientation of eyes and head compared to the use of pointing). In this way, we were able to differentiate more structurally closer items on the photos than on the videos.

The AOI created with the mean shift clustering method are different depending on group and stimulus. In addition, the Voronoi method (Over et al., 2006) for creating AOI made it possible to compare data from different groups. Thus, the use of *a posteriori* AOI helped us to avoid reaching conclusions about our data by arbitrarily separating fixations into areas, to match our hypotheses. For example, we realized that, with our stimuli, it is impossible to distinguish between and compare fixations on the eyes and the mouth. The specific exploration style of children with ASD has often been studied using eye-tracking (Jones et al., 2008; Chawarska et al., 2013). However, it is difficult to compare our results with those obtained by Shic et al. (2014), who showed that children with ASD were less likely to fixate the actress's eyes in a video in which she was speaking motherese, compared to other types of stimuli (images and videos without language). Likewise, we cannot compare our results with those of Saitovitch et al. (2013), who found that different kinds of stimuli (photos and videos) did not have a significant differential impact on fixation on the eyes and mouth.

This study has revealed similarities between children with ASD and typically developing children. All groups looked longer at the face, showing a real interest in this social factor in the joint attention scene (Chawarska et al., 2013). We also noted that they all show more interest in the target, which they look at longer than the background. However, in the literature, there is often a strong interest in the background among ASDs (Riby and Hancock, 2008; Chawarska and Shic, 2009). Perhaps this interest depends on the definition of background. Usually, “background” refers to fixations that do not fall within the predefined AOI. Of course, the smaller and more precise the AOI are, the more data points there are corresponding to the background. On the other hand, with our method, we defined background fixations as a function of the data density in certain very precise areas of the stimulus.

Nevertheless, differences were observed between groups: ASD children looked at the stimuli for a shorter period of time than TCs but there is no difference between ASDs and TDs. Stimuli seem less interesting for children with a lower level of communicative development in ASDs or TDs groups. Moreover, we observed that control group children looked for less time at the video than at the photos while there is no difference in ASDs. This result is contrary to our predictions. We actually expected less difference in visual exploration between ASDs and control children when confronted with videos. We hypothesized such lack of difference based on works on movement interest that have been found in studies of biological motion in typically developing children (Johansson, 1973), whereas other studies highlighted a change in the visual preference for biological motion in children with ASD (e.g., Klin et al., 2009; Falck-Ytter et al., 2013a). Moreover, the study by Pierce et al. (2011) showed that children with ASD are more attracted by movements of geometric shapes than by human movements.

Furthermore, our results might be also linked to the social implication underlying our joint attention scene. The video implicitly asks the children to be part of an interaction with the actress who is actually speaking to them. Therefore, while the photograph is generally viewed more passively, following the social dynamics involved in a video requires a greater degree of social competence (Yeates et al., 2007; Nader-Grosbois, 2011). This is why we considered our video stimuli to be more ecological than a photograph but, according to Hanley et al. (2013), a static photograph showing a facial expression (where such an expression would be the cue of a social interaction) could be linked to an ecological social situation. Thus, the absence of difference in visual exploration between static and dynamic stimuli for ASD children could be explained by the ecological aspect of the photographs.

About target fixation, we observe an effect of the cue type. In video stimuli, the only difference observed for children with autism concerns the use of pointing in addition to the orientation of the head compared to the eyes orientation toward the target. There is no effect of verbalizations. The bimodal nature of dynamic stimuli that implies sound and movement may explain ASD result. In fact, bimodal dynamic stimuli require more attentional resources. Chawarska et al. (2013) showed that children with ASD lost interest in a video stimulus when it featured verbalizations addressing them. On the other hand, TD children look at the target longer when the actress points, compared to head orientation or head orientation with pointing and verbalizations. Benjamin et al. (2014) showed that preschoolers are more interested in the target when the actor points in addition to orienting his head, but naming the target while looking at it has no effect on how long children explore it. In TC children, pointing or pointing and verbalizing results in more target gazes compared to eye or head orientation situations. It therefore seems that the cues do not have the same effect depending on the developmental and chronological age of children.

Interestingly, there are more similarities between ASD and control groups when they look at photos. In this perceptually uni-modal context, where we do not directly observe the pointing movement but only the resulting pointing, the stillness of the photograph allows all children to spend a longer time looking at the target compared to the photograph where the actress does not point at the target but orients her head or eyes toward the target. The photo representing the scene where the actress looks at the target, points at it, and verbalizes by saying “oh look” does not really present sound verbalizations. Therefore, it does not elevate it to a higher status than the photo without the open mouth as a sign of verbalization. In a future study, it would be interesting to compare data on videos without speech, that is, where an actress looks at the target and orients her head as compared to a scene in which she talks while she makes the same actions.

Finally, we can say that the use of videos in research and clinical settings is valuable. Indeed, when the development of children with ASD is being monitored, it is essential to obtain visual information. For example, therapists often say “look at me” when they want to work on joint attention skills (Barthélémy et al., 1995; Rogers and Dawson, 2013). The study by Rudy et al. (2014) also showed the value of video tutorials to teach the gaze-tracking skills required for joint attention. This is, of course, impossible with still photos. Future studies could focus on imitation behaviors displayed by ASD children when watching an explanatory video of a joint attention exercise. This is precisely what we observed during our research sessions. In addition, videos can be used to slow down movement in tutorials (Gepner, 2001; Gepner et al., 2010). For example, eye-tracking studies have shown that fixations are longer and focus more on the face when the pace of a story told by a narrator is slowed down by 50% (Tardif et al., 2016; Charrier et al., 2017).

## Conclusion

The aim of this study was to identify differences between groups in studying joint attention with two kinds of stimuli: static and dynamic. The theoretical and practical implications of this study concern, on the one hand, the use of stimuli in research and, on the other hand, the use of didactic stimuli in practice. The use of a method to create AOI *a posteriori* enabled us to limit overhasty conclusions on differences in visual exploration. The results show the value of both kinds of stimuli. Photos allow for the *a posteriori* creation of more precise AOI. Videos highlight the singular nature of visual exploration of this joint attention scene by children with ASD compared to control children. The characteristic exploration of the joint attention scene in both kinds of stimuli raises questions regarding how children with ASD understood this task. Furthermore, the fact that photo stimuli reduce the social aspect of the scene implies a similar exploration between ASD and typical children. Researchers must therefore ask themselves questions when creating experimental tasks. On the one hand, photos allow a similar exploration in all children; on the other hand, videos are more naturalistic and can be used in a didactic practice. Given their complementary nature, both kinds of stimuli can be used to investigate theoretical questions regarding joint attention in children with ASD. Still, videos are preferable in clinical practice, because they can be used to teach social skills to children with ASD (Rudy et al., 2014; Kourassanis-velasquez and Jones, 2018). Finally, since joint attention depends on an interactive dynamic, it seems clear that the use of videos with eye-tracking will allow for better responses to researchers' theoretical questions about social cognition, for example, regarding the linearity of gaze in studies that compare gaze tracking with or without head orientation (Riby and Doherty, 2009; Bedford et al., 2012).

## Limitations and Propsects for Future Research

There are several limitations affecting this study. On the one hand, we note that we used an innovative method compared to the classical literature using algorithms provided by eye-tracker manufacturers. However, the mean shift clustering algorithm that we adapted for our study was developed by Einbeck (2011), a researcher in mathematics. In addition, it appears that using a prerecorded stimulus depicting a joint attention scene does not enable one to fully grasp children's understanding of this task. Regarding the comparison of stimuli with and without sound, in correspondence with the rest of the protocol, we chose not to make children listen to music while they saw the photos. Nevertheless, comparing videos with sound and obvious movement to photos that by nature are soundless and present a static image remains very debatable.

This work suggests several research avenues. For example, it would be interesting to investigate visual exploration using other kinds of stimuli. Thus, we could not only compare photo and video data, but also work with live interactions using more sophisticated data analysis methods than gaze-path coding, like Thorup et al. (2016). In addition, in the context of joint attention, it would be interesting to assess the impact of a joint attention bid by an avatar or robot, as opposed to a human being. Recent studies have shown that these new interaction partners can be valuable in teaching joint attention skills such as gaze tracking and looking at the partner (David et al., 2017; Tapus et al., 2018).

## Ethics Statement

This study did not require authorization by an ethics committee, based on the recommendations for psychological research in France (https://www.legifrance.gouv.fr/eli/decret/2017/5/9/AFSP1706303D/jo/texte). Nevertheless, our CNIL (Commission nationale de l'informatique et des libertés: National Commission for Informatics and Liberties) declaration number of research conformity is: 2208663 v 0. After reading the objectives of this study, the children's legal representatives provided informed consent in accordance with the recommendations of the Declaration of Helsinki of June 1964 (amended at the 64th General Assembly of the World Health Organization in October 2013).

## Author Contributions

All authors listed have made a substantial, direct and intellectual contribution to the work, and approved it for publication.

### Conflict of Interest

The authors declare that the research was conducted in the absence of any commercial or financial relationships that could be construed as a potential conflict of interest.
